# Whey protein hydrolysate mitigates both inflammation and endotoxin tolerance in THP‐1 human monocytic leukemia cells

**DOI:** 10.1002/iid3.737

**Published:** 2022-11-07

**Authors:** Fuka Ishikawa, Takeshi Matsubara, Takahiro Koyama, Hiroshi Iwamoto, Kazuhiro Miyaji

**Affiliations:** ^1^ Health Care & Nutrition Science Institute R&D Division, Morinaga Milk Industry Co. Ltd. Kanagawa Zama Japan

**Keywords:** endotoxin tolerance, immunosuppression, inflammation, whey protein hydrolysate

## Abstract

**Introduction:**

It is important to control both inflammation and immunosuppression after severe insults, such as sepsis, trauma, and surgery. Endotoxin tolerance is one of the immunosuppressive conditions and it has been known that endotoxin tolerance relates to poorer clinical outcomes in patients with severe insults. This study investigated whether whey protein hydrolysate (WPH) mitigates inflammation and endotoxin tolerance in THP‐1 human monocytic leukemia cells.

**Methods:**

Endotoxin tolerance can be experimentally reproduced by two consecutive stimulations with lipopolysaccharide (LPS). THP‐1 cells were incubated with LPS and WPH (first stimulation). After collecting the culture supernatant to evaluate the effect on inflammation, the cells were washed and restimulated by 100 ng/ml LPS (second stimulation). The culture supernatant was again collected to evaluate the effect on endotoxin tolerance. Concentrations of LPS and WPH in the first stimulation were adjusted to evaluate their dose dependency. Cytokine levels in the supernatant were determined by enzyme‐linked immunosorbent assay. Statistical analysis was performed using the student's *t*‐test or Dunnett's test.

**Results:**

Five mg/ml WPH significantly decreased interleukin (IL)‐6 (*p* = .006) and IL‐10 (*p* < .001) levels after the first LPS stimulation (1000 ng/ml). WPH significantly increased tumor necrosis factor‐alpha (*p* < .001) and IL‐10 (*p* = .014) levels after the second LPS stimulation. The suppressive effect of WPH on inflammation and endotoxin tolerance was dependent on the concentrations of LPS and WPH. The effective dose of WPH for endotoxin tolerance was lower than its effective dose for inflammation.

**Conclusion:**

WPH mitigated both inflammation and endotoxin tolerance. Therefore, WPH might be a candidate for valuable food ingredients to control both inflammation and immunosuppression after severe insults.

## Graphical abstract

Endotoxin tolerance and inflammation were assessed in THP‐1 cells. Whey protein hydrolysate suppressed lipopolysaccharide (LPS)‐induced inflammation. Whey protein hydrolysate mitigated LPS‐induced endotoxin tolerance.

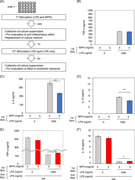

## 1 INTRODUCTION

Severe inflammation is induced by many clinical conditions that include surgery, sepsis, severe burns, acute pancreatitis, and trauma. These conditions can induce multiple organ failures.^1^ Therefore, controlling the inflammatory response is important to prevent death caused by multiple organ failure.^2^ Improved treatment has decreased the incidence of hyperinflammation in patients. However, patients can subsequently experience an immunosuppressive phase.^3^ For example, most septic patients are in a state of immunosuppression and die from opportunistic infections.^4^, ^5^

Endotoxin tolerance is one of the immunosuppressive conditions.^6^ Endotoxin tolerance refers to a reduction in response to endotoxins after various inflammatory conditions, such as sepsis, trauma, and surgery.^7^, ^8^ Recently, it has been reported that the major immunologic abnormality in coronavirus disease 2019 (COVID‐19) is a profound defect in host immunity including T cell exhaustion and monocyte endotoxin tolerance,^9^ and that endotoxin tolerance is more severe in critical ill patients with COVID‐19 than those without COVID‐19.^10^ Endotoxin tolerance can be experimentally reproduced by two consecutive stimulations with lipopolysaccharide (LPS).^11^ Monocytes and macrophages are important in the development of endotoxin tolerance.^12^, ^13^ Monocytes from patients with sepsis, who are known to be in the state of endotoxin tolerance, produce less pro‐inflammatory cytokines, such as tumor necrosis factor‐alpha (TNF‐α), interleukin (IL)‐12, IL‐23, and IL‐6, in response to LPS stimulation, compared to monocytes from healthy volunteers.^11^ Heagy et al.^14^ showed that the low levels of cytokine production from the whole blood of patients admitted to the intensive care unit stimulated by LPS *ex vivo* are associated with poor clinical outcomes, including high mortality. The experimental approach that uses LPS stimulation has also been applied to the THP‐1 human monocytic leukemia cell line. Endotoxin tolerance was assessed in THP‐1 cells based on the reduction in TNF‐α expression or production.^11^, ^15^, ^16^, ^17^ Following severe insults, it is important to control both inflammation and immunosuppression. In an experiment in which monocytic cells from healthy volunteers were stimulated twice by LPS, the first LPS stimulation correlated inversely with TNF‐α levels after the second LPS stimulation.^18^ These findings imply that the severity of the initial inflammation is associated with the severity of the ensuing immunosuppression.

Whey protein hydrolysate (WPH) and peptides derived from whey protein have various functions demonstrated by in vivo and in vitro experiments, such as anti‐inflammation,^19^, ^20^, ^21^ antihypertensive,^22^ improving glucose^23^ and lipid metabolism,^24^ and strengthening skin barrier.^25^, ^26^ In regard to anti‐inflammatory effect, Tavares et al.^19^ confirmed that oral administration of WPH prevents inflammation via prostaglandin system under the paw edema test in mice. Kume et al.^20^ reported that an immune‐modulating diet containing whey peptides suppresses bacterial translocation and inflammatory cytokine levels in the rat model of small bowel disorders. Moreover, Ma et al.^21^ demonstrated peptides derived from whey protein reduce expression of inflammatory cytokine in RAW 264.7 mouse macrophage cells. In this study, we hypothesized that suppression of inflammation by WPH could mitigate subsequent endotoxin tolerance and investigated this hypothesis in THP‐1 cells.

## 2 MATERIALS AND METHODS

### 2.1 Preparation of WPH

WPH was obtained from Morinaga Milk Industry (Tokyo, Japan). The degree of hydrolysis of the WPH was 16%. WPH was dissolved at 20 mg/ml in RPMI 1640 (Gibco) supplemented with 1% penicillin‐streptomycin (Gibco). LPS contained in WPH was removed by centrifugation using an Amicon Ultra‐15 100 K centrifugal filter (Merck Millipore). Endospecy ES‐50M set (Seikagaku) was used according to the manufacturer's protocol to confirm LPS removal.　The fetal calf serum (FCS) concentration of the WPH sample was adjusted to 10%.

### 2.2 Cell culture and stimulation

THP‐1 human monocytic leukemia cells obtained from KAC were cultured in RPMI 1640 containing 10% FCS (Cell growth culture No. 103; KAC) supplemented with 1% penicillin‐streptomycin (Gibco). The cells were maintained at a density of <1.0 × 10^6^ cells/ml at 37°C in a 5% CO_2_ incubator.

**Figure 1:**
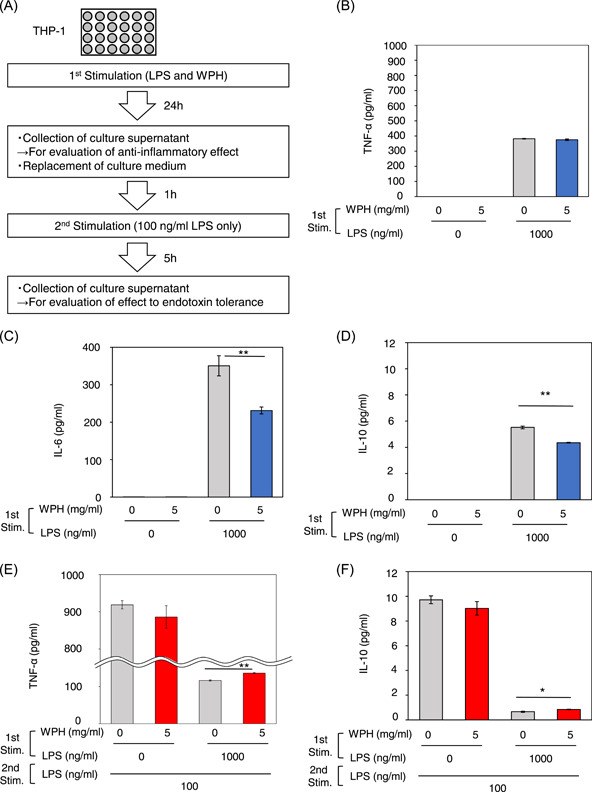
Whey protein hydrolysate (WPH) reduces the production of interleukin (IL)‐6 and IL‐10 after the first lipopolysaccharide (LPS) stimulation and increases the production of tumor necrosis factor (TNF)‐α and IL‐10 after the second LPS stimulation. (A) Schematic representation of the experimental procedure. (B–F). Cells were not stimulated or stimulated by 5 mg/ml WPH or 1000 ng/ml LPS, followed by restimulation with 100 ng/ml LPS. The levels of (B) TNF‐α, (C) IL‐6, and (D) IL‐10 after the first LPS stimulation, and (E) TNF‐α and (F) IL‐10 after the second LPS stimulation were measured. Three independent experiments were performed. Similar results were obtained and the representative result was represented. Values are presented as mean ± SEM　(*n* = 4). *p* < .05 and 0.01 are indicated by * and **, respectively, compared to 0 mg/ml WPH in the same LPS concentration. Stim, stimulation.

The schematic experimental procedure is shown in Figure 1A. The cells were seeded onto 24‐well plates at a density of 2.5 × 10^5^ cells/well and incubated for 24 h (*n* = 3–4). The cells were then treated with WPH and LPS from *Escherichia coli* O111:B4 (L4391; Sigma‐Aldrich) and incubated for 24 h (first LPS stimulation). The concentrations of WPH and LPS were adjusted for each experiment and are described in the figure legends. Following incubation, culture supernatants were collected, and the cells were washed twice with the medium and reseeded. Following 1 h of incubation, the cells were restimulated with 100 ng/ml LPS for 5 h (second LPS stimulation). This culture supernatant was also collected. All culture supernatants were stored at −80°C.

To investigate whether the effect of WPH was dependent on the LPS concentration during the first stimulation, cells were stimulated by 0, 10, 100, or 1000 ng/ml LPS under the condition of 0 or 5 mg/ml WPH, followed by restimulation with 100 ng/ml LPS. In evaluating the dose dependency of WPH, cells were stimulated by 0 or 100 ng/ml LPS under the condition of 0, 0.5, 1, 2, or 5 mg/ml WPH, and followed by restimulation with 100 ng/ml LPS.

### 2.3 Measurement of cytokines

The levels of IL‐6, IL‐10, and TNF‐α in the supernatants were determined using the following　commercial enzyme‐linked immunosorbent assay (ELISA) kits according to the manufacturer's protocol; IL‐6 Human Uncoated ELISA Kit (88‐7066‐88), IL‐10 Human Uncoated ELISA Kit (88‐7106‐88), and TNF alpha Human Uncoated ELISA Kit (88‐7346‐88) (all from Invitrogen).

### 2.4 Statistical analyses

Data are presented as the mean ± standard error of the mean (SEM) and were analyzed using JMP version 13.2.1 (SAS Institute). The student's *t*‐test was used. In the results of the experiment to determine the dose dependency of WPH, Dunnett's test was used for multiple comparisons between THP‐1 cells received with first LPS stimulation. In Dunnett's test, the cells without WPH during first LPS stimulation was used as control. Statistical significance was set at *p* < .05.

## 3 RESULTS

### 3.1 WPH reduces IL‐6 and IL‐10 production after the first LPS stimulation and increases TNF‐α and IL‐10 production after the second LPS stimulation

The anti‐inflammatory effect is often assessed by changes in cytokine levels in LPS‐stimulated cells.^21^ To investigate whether WPH has anti‐inflammatory effects, we determined the levels of TNF‐α, IL‐6, and IL‐10 after the first LPS stimulation. No difference in TNF‐α levels between cells with and without WPH was evident (Figure 1B). However, IL‐6 (*p* = .006) and IL‐10 (*p* < .001) levels in the cells incubated with WPH were significantly lower than in those without WPH incubation (Figure 1C,D).

Endotoxin tolerance is assessed by a decrease in TNF‐α production after a second LPS stimulation.^11^ To investigate whether WPH can mitigate endotoxin tolerance, the levels of TNF‐α and IL‐10 after the second LPS stimulation were determined. TNF‐α levels in the cells that received the first LPS stimulation were clearly lower than those from cells that did not receive LPS stimulation (Figure 1E). This indicates that the first LPS stimulation for 24 h induced endotoxin tolerance. In the cells that received the first LPS stimulation, the levels of TNF‐α (*p* < .001) and IL‐10 (*p* = .014) after the second LPS stimulation were significantly higher from cells incubated with WPH compared to cells without WPH addition (Figure 1E,F).

These collective results indicate that WPH reduced the levels of IL‐6 and IL‐10 after the first LPS stimulation and increased TNF‐α and IL‐10 production after the second LPS stimulation.

### 3.2 WPH‐mediated mitigation of inflammation and endotoxin tolerance is dependent on the LPS dose during the first stimulation

We next investigated whether the suppressive effect of WPH on inflammation and endotoxin tolerance was dependent on the LPS dose during the first stimulation.

**Figure 2:**
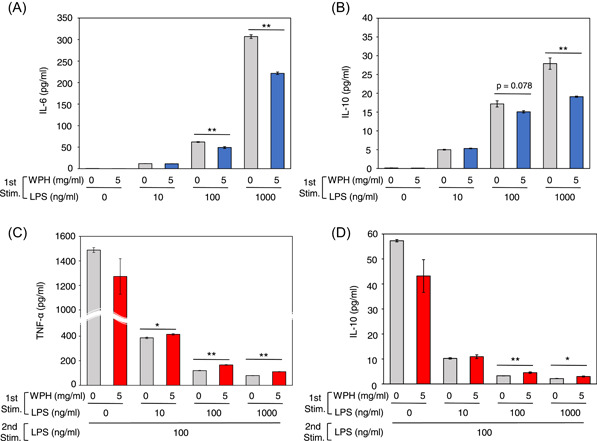
Whey protein hydrolysate (WPH) mitigation of inflammation and endotoxin tolerance are dependent on lipopolysaccharide (LPS) dose in the first LPS stimulation. (A–D) Cells were not stimulated or stimulated by 5 mg/ml WPH or 10, 100, or 1000 ng/ml LPS, followed by restimulation with 100 ng/ml LPS. The levels of (A) interleukin (IL)‐6 and (B) IL‐10 after the first LPS stimulation, (C) tumor necrosis factor (TNF)‐α, and (D) IL‐10 after the second LPS stimulation were measured. Three independent experiments were performed. Similar results were obtained and representative result was represented. Values are presented as mean ± SEM (*n* = 3). *p* < .05 and .01 are indicated by * and **, respectively, compared to 0 mg/ml WPH within the same dose LPS in the first stimulation. Stim, stimulation.

After the first stimulation of THP‐1 cells with 100 and 1000 ng/ml LPS, the IL‐6 levels in cells incubated with WPH were significantly lower (*p* = .006 and *p* < .001, respectively) than that in cells without WPH incubation (Figure 2A). The IL‐10 levels from cells incubated with WPH were significantly lower after 1000 ng/ml LPS stimulation (*p* = .004) and tended to be lower after 100 ng/ml LPS stimulation (*p* = .078) than that from cells incubated without WPH (Figure 2B). However, there was no difference in the IL‐6 and IL‐10 levels after the first stimulation with 10 ng/ml LPS between the cells incubated with and without WPH (Figure 2A,B).

When cells were treated with 10, 100, and 1000 ng/ml LPS as the first stimulation, (*p* = .035, *p* < .001, and *p* < .001, respectively) TNF‐α levels after the second LPS stimulation from the cells incubated with WPH during the first stimulation were significantly higher than that from the cells incubated without WPH (Figure 2C). Furthermore, when cells were treated with 100 and 1000 ng/ml LPS as the first stimulation (*p* = .0097 and *p* = .013, respectively), IL‐10 levels after the second LPS stimulation from the cells incubated with WPH were significantly higher than those from the cells incubated without WPH (Figure 2D). These results indicate that WPH reduced the production of IL‐6 and IL‐10 after the first LPS stimulation and increased the production of TNF‐α and IL‐10 after the second LPS stimulation, depending on the dose in the first LPS stimulation.

### 3.3 Dose dependency of WPH in mitigating inflammation and endotoxin tolerance

To further characterize the effects of WPH on inflammation and endotoxin tolerance, we investigated the dose dependency of WPH.

**Figure 3:**
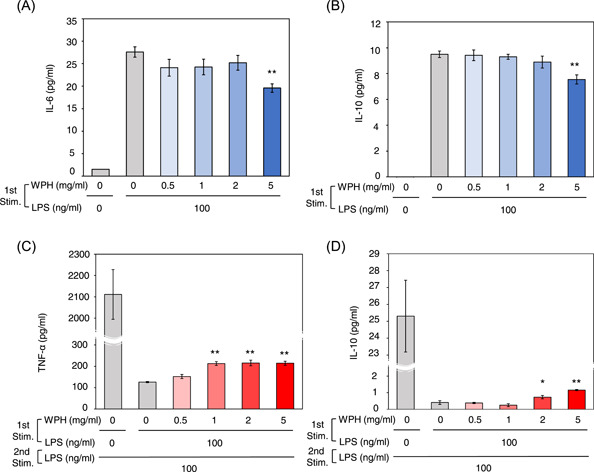
Dose dependency of whey protein hydrolysate (WPH). (A–C) Cells were not stimulated or stimulated by 0.5, 1, 2, or 5 mg/ml WPH, or 100 ng/ml lipopolysaccharide (LPS), followed by restimulation with 100 ng/ml LPS. The levels of (A) interleukin (IL)‐6 and (B) IL‐10 after the first LPS stimulation and (C) tumor necrosis factor (TNF)‐α and (D) IL‐10 after the second LPS stimulation were measured. Three independent experiments were performed. Similar results were obtained and the representative result was represented. Values are presented as mean ± SEM (*n* = 4). *p* < .05 and 0.01 are indicated by * and **, respectively, compared to cells stimulated by 100 ng/ml LPS in first LPS stimulation and without WPH. Stim, stimulation.

After the first LPS stimulation, the levels of IL‐6 (*p* = .007) and IL‐10 (*p* = .005) in the cells incubated with 5 mg/ml WPH were significantly lower than those in cells incubated without WPH (Figure 3A,B). However, the levels of IL‐6 and IL‐10 after the first stimulation of cells incubated with 0.5, 1, and 2 mg/ml WPH did not change from the cells incubated without WPH (Figure 3A,B).

After the second LPS stimulation, the TNF‐α level in the cells incubated with 1, 2, and 5 mg/ml WPH, but not 0.5 mg/ml WPH, during the first stimulation was significantly (*p* < .001 for all comparisons) higher than that from the cells incubated without WPH (Figure 3C). After the second LPS stimulation, the IL‐10 level in cells incubated with 2 and 5 mg/ml WPH (*p* = .041 and *p* < .001, respectively), but not at 0.5 and 1 mg/ml WPH, was higher than that from the cells incubated without WPH (Figure 3D). These results indicate that WPH decreased IL‐6 and IL‐10 production after the first LPS stimulation at 5 mg/ml, increased TNF‐α production after the second LPS stimulation at 1, 2, and 5 mg/ml, and increased IL‐10 production after the second LPS stimulation at 2 and 5 mg/ml.

## 4 DISCUSSION

Patients experiencing insults, such as surgery, sepsis, or trauma, can become immunosuppressed concomitant with or sometime after the initial inflammation.^6^, ^8^ Endotoxin tolerance is one of the causes of immunosuppression.^6^ In this study, we demonstrated that WPH mitigates inflammation and subsequent endotoxin tolerance in THP‐1 cells.

After insult, various cytokines are released into circulation. The levels of the IL‐6 inflammatory cytokine, one of the biomarkers of trauma and sepsis, in patients with sepsis are correlated with the severity of organ failure.^27^ Consistent with previous studies showing anti‐inflammatory effects of fermented milk^28^ and a diet that includes whey peptides,^20^ WPH reduced IL‐6 production after the first LPS stimulation in the present study. TNF‐α is an important pro‐inflammatory cytokine.^29^ High TNF‐α levels are associated with mortality in patients with sepsis.^30^ In this study, WPH did not affect TNF‐α levels after the first stimulation. These findings differ from that of Ma et al.,^21^ who reported that peptides isolated from whey protein reduced TNF‐α levels in RAW 264.7 macrophage cells. In THP‐1 cells, a study reported that TNF‐α levels peaked 4 h after LPS stimulation, while IL‐6 levels peaked after 12 or 24 h.^31^ Therefore, the different effects of WPH on TNF‐α and IL‐6 levels could have occurred because of the time point when the supernatants were collected. In the present study, WPH decreased the level of the anti‐inflammatory cytokine IL‐10 after the first LPS stimulation. A previous study reported that IL‐10 was produced in response to insult, and the overproduction of IL‐10 was associated with severity and fatal outcome in patients with sepsis.^32^ Therefore, reducing IL‐10 may have a positive effect on at least these patients.

Endotoxin tolerance is characterized by a reduction in response to endotoxin^7^, ^8^ and is often assessed by a decrease in TNF‐α production or expression in response to successive LPS stimulation.^11^, ^17^ Previous in vitro studies have shown that the ability of IL‐10 production in response to LPS stimulation is decreased due to endotoxin tolerance.^33^, ^34^ Moreover, low IL‐10 production after ex vivo LPS stimulation of whole blood from patients with sepsis may be related to persistent organ dysfunction.^35^ In this study, after the second LPS stimulation, the levels of TNF‐α and IL‐10 from cells treated with the first LPS stimulation were clearly decreased compared to those from cells not treated with the first LPS stimulation, and WPH inhibited the decreases in the production of TNF‐α and IL‐10. These results indicate that THP‐1 cells acquired endotoxin tolerance, which could be mitigated by WPH. Endotoxin tolerance is regarded as a necessary mechanism for self‐protection.^8^ For example, mice prestimulated with a low dose of LPS displayed a reduced death rate upon restimulation with a lethal dose of LPS.^36^ In humans, consecutive LPS administrations reportedly attenuated the fever response.^37^ Meanwhile, lower cytokine production when blood was stimulated by LPS ex vivo, that is endotoxin tolerance, was related to poorer clinical outcomes in patients with trauma, sepsis, or in the intensive care unit.^14^, ^38^, ^39^ Recently, endotoxin tolerance has also been regarded as an important issue in COVID‐19,^9^, ^10^ Furthermore, the direct relationship between mortality and the severity of endotoxin tolerance was demonstrated in the animal model with a severe polymicrobial intra‐abdominal infection.^40^ These findings concerned with endotoxin tolerance have attracted clinical attention. Although endotoxin tolerance has two different aspects, we focused on the latter (i.e., an unlikable effect on clinical outcomes in patients with severe insults) and demonstrated the suppression of endotoxin tolerance by WPH. Previous studies have demonstrated that the sirtuin‐1 inhibitor and capric acid mitigated endotoxin tolerance in THP‐1 cells.^15^, ^16^ Other substances may be identified in future studies.

Some studies have investigated whether cytokines produced during initial inflammation are associated with endotoxin tolerance development. Preincubation with IL‐10 or transforming growth factor‐beta (TGF‐β), neither TNF‐α nor IL‐6, partially led to endotoxin tolerance in monocytic cells.^18^, ^41^ Randow et al.^41^ demonstrated that IL‐10 and TGF‐β produced during initial inflammation are necessary to lead to endotoxin tolerance using neutralizing antibodies. However, the need for IL‐10 to develop endotoxin tolerance is controversial given the observation that IL‐10 knockout mice develop endotoxin tolerance.^42^ In this study, WPH decreased IL‐10 levels after the first LPS stimulation only when the first LPS dose was higher than 100 ng/ml. However, WPH inhibited the reduced level of TNF‐α after the second LPS stimulation when the first LPS dose was 10–1000 ng/ml. Furthermore, the effective WPH dose to mitigate endotoxin tolerance was lower than that which affected IL‐10 levels during initial inflammation. Thus, WPH affected endotoxin tolerance, even if it did not influence IL‐10 levels produced during initial inflammation. These findings are not entirely consistent with our hypothesis that suppression of inflammation by WPH can mitigate the subsequent endotoxin tolerance and suggest that WPH could affect inflammation and endotoxin tolerance through different mechanisms. However, there is a possibility that anti‐inflammatory effects were not detectable because we evaluated inflammation and endotoxin tolerance only by ELISA‐determined cytokine levels. Additionally, it was difficult to detect TGF‐β production in our experimental procedure because the cell culture medium contained TGF‐β. To overcome these limitations, further analyses are necessary. These include the detection of mRNA expression. LPS activates Toll‐like receptor (TLR)‐4 and its downstream signaling, including nuclear factor‐κB (NF‐κB) pathway or mitogen‐activated protein kinase pathway. This signaling is closely related to endotoxin tolerance.^8^, ^11^, ^43^, ^44^ It has been reported that IL‐1 receptor‐associated kinase (IRAK) 3, which is mainly found in macrophages and monocytes, inhibits the signaling from TLR‐4 to downstream NF‐κB and reduces cytokine production such as TNF‐α.^11^, ^44^ Very recently, meta‐analysis reported that increase of IRAK3 expression is related to the reduction of TNF‐α expression after second stimulation by LPS or other TLRs agonist in vivo and in vitro.^43^, ^44^ Further study about these signaling pathways is necessary to clarify more detailed mechanisms for mitigating endotoxin tolerance by WPH.

This study is significant from the perspective that WPH can be a candidate food ingredient to control inflammation and immunosuppression after severe clinical insults, such as surgery, sepsis, severe burns, and trauma. Like these clinical conditions, since patients with severe COVID‐19 suffer from systemic inflammation and subsequent immunosuppression, treatment to enhance or suppress immune response should be applied based on the immune status.^45^ This implicates that it is important to control both inflammation and immunosuppression after severe clinical insults. However, this study has several limitations. First, this study did not consider the digestion and absorption of WPH and the blood level of WPH. Second, since THP‐1 is a cell line and may be less sensitive to LPS and/or other substances such as capric acid than peripheral blood mononuclear cells,^16^, ^46^ THP‐1 cells were treated in this study with high‐concentration of WPH, which is thought to be over a physiological concentration range. Third, although we observed that WPH inhibited the reduction of TNF‐α and IL‐10 after the second stimulation, it could not restore the levels of these cytokines to those in cells not exposed to the first LPS stimulation. To solve these limitations related to the clinical efficacy of WPH, the physiological efficacy of WPH needs to be investigated ex vivo or in vivo, such as in animal tests and/or clinical tests. Forth, we did not evaluate whether WPH neutralizes LPS. It is known that specific peptides such as lactoferricin, which is derived from lactoferrin of whey protein hydrolyzed by pepsin, can bind to LPS and suppress inflammation.^47^ However, WPH may contain few lactoferricin because the raw material of WPH, whey protein concentrate, contains few lactoferrin. Finally, only a single type of WPH was used. Kiewiet et al.^48^ demonstrated that the effects of protein hydrolysates on cytokine production are dependent on the degree of hydrolysis. It is necessary to identify the fraction or sequence of bioactive WPH peptides and to investigate whether other WPHs, whose degree of hydrolysis differs from current WPH, can mitigate inflammation and endotoxin tolerance.

## 5 CONCLUSION

We demonstrated that WPH mitigates both inflammation and endotoxin tolerance. This mainly agrees with our hypothesis that suppression of inflammation by WPH can mitigate endotoxin tolerance. However, there are some exceptions, such as the effective dose of WPH on endotoxin tolerance is lower than the effective dose on inflammation. To the best of our knowledge, this is the first study to report the simultaneous efficacy of a single substance on both inflammation and endotoxin tolerance. Further detailed studies are required to clarify the relationship between inflammation and endotoxin tolerance.

## AUTHOR CONTRIBUTIONS

All authors have read and approved the final manuscript. Fuka Ishikawa prepared the original draft. Fuka Ishikawa and Takeshi Matsubara performed the research, analyzed the data, and wrote the paper. Takeshi Matsubara designed the research study. Takahiro Koyama, Hiroshi Iwamoto, and Kazuhiro Miyaji contributed to essential reagents and tools and supervised the study.

## CONFLICTS OF INTEREST

All authors are employees of Health Care & Nutrition Science Institute, R&D Division, Morinaga Milk Industry Co., Ltd. (Tokyo, Japan). This research did not receive any specific grant from funding agencies in the public, commercial, or not‐for‐profit sectors.

## Data Availability

The data used to support the findings of this study are available from the corresponding author on reasonable request.
